# Review of monitoring systems for stored grains in a modified atmosphere

**DOI:** 10.1016/j.heliyon.2025.e42347

**Published:** 2025-01-30

**Authors:** Louis Labrot–Rhodes, Eric Campo, Pierre Poujaud

**Affiliations:** a LAAS-CNRS, Toulouse University, CNRS, INSA, UT2J, Toulouse, France; b NOX Storage SAS, Villemur-sur-Tarn, France

**Keywords:** Grain storage, Monitoring technology, Modified atmosphere, CO_2_ conservation, IoT

## Abstract

Losses during grain storage have become unacceptable owing to the growing needs of the world's population and climate problems. Monitoring systems in combination with measured data analytics can help to prevent insect or mold damage to stored grains, whatever the storage technique used. Indeed, it is necessary to identify and understand the risks associated with storage and the quantities available to ensure optimal storage when assessing the relevance of solutions. First, we discuss and compare existing monitoring solutions. Next, we examine smart agriculture approaches under development for post-harvest monitoring of warehouses or silos, which is currently well covered by solutions proposed by companies or research laboratories. However, the comprehensive review indicates that there is currently a lack of solutions for monitoring grains stored in hermetic bags in modified atmospheres, particularly those enriched with carbon dioxide. This solution seems interesting because it does not use any phytosanitary products and has proven its effectiveness. Different works on the development of these monitoring solutions are presented. Finally, the various challenges raised are discussed.

## 1 Introduction

The growing population and amount of meat consumed per capita in some countries have led to an increasing demand for agricultural production; thus, there is a need to increase the yields per hectare of cultivated land or the area under cultivation to meet the increasing demand [1]. However, it is not sufficient to only increase production; it is necessary to also be able to store the crops to ensure their availability throughout the year and well beyond harvest to avoid losses. Several studies have reported that reducing losses can greatly limit food insecurity and greenhouse gas emissions [1,2]. Food wastage attributed to consumers and in the supply chain is responsible for 6 % of the global greenhouse gas emissions [3]. According to the Food and Agriculture Organization of the United Nations (FAO), the number of undernourished people in the world increased in 2020, and even before the COVID-19 pandemic, it had already reached 720–811 million people [4]. Thus, food loss is being closely monitored by world leaders and researchers [1,5]. The calculations of food loss can yield different figures depending on the assumptions considered [1]; however, approximately one-third of the world's agricultural production is lost every year [5,6]. Some calculations consider losses after storage and others from agricultural production (considering losses during harvesting, sorting, etc.). The percentage of loss can correspond to the mass or calories lost [2,6]. Among the four categories (fruits and vegetables, oilseeds and pulses, roots and tubules, and cereals), cereals are the primary source consumed and provide 63 % of the total food supply (after losses and waste); therefore, they account for 57 % of the total losses (in terms of calories in the years 2005–2007). The second category most represented in losses is “oilseeds and pulses,” and thus, approximately three-quarters of the world's losses are from cereals, oilseeds, or pulses [2]. The focus of this article is on the storage of cereals, legumes, and oilseeds, so the generic term “grain” is used for simplicity.

The presence of live insects can lead to serious consequences for rejecting the entire grain lot, which results in a significant economic loss to the grain supplier [7]. Further, methods and technologies have been developed to prevent grain loss in modified atmospheres, cold rooms, and/or monitoring. A modified atmosphere is a generic term that encompasses all cases wherein the composition of the atmosphere is modified to regulate insects and to maintain product quality. Although modified atmosphere storage has been used for thousands of years, advances in flexible and hermetic materials have driven growing interest in the durable storage of agricultural products [8]. Stored food deteriorates when environmental conditions are unfavorable, which can promote the development of insects and molds, and decrease germination power. Therefore, it is necessary to monitor stored grains to implement corrective measures when conditions deteriorate (increase in temperature, humidity, insect number, or change in the composition or pressure of the storage atmosphere in the case of modified atmosphere storage). There have been no reviews of grain storage monitoring systems and the only reviews we found (2016–2023) were few scientific references cited in publications that proposed a system for grain-storage monitoring. These “state-of-the-art” sections often ignored commercially available systems and focused only on few scientific publications. Therefore, in this review, the aim of this article is to provide illustrative publications and industry products of technological solutions for grain storage and to compare them. For commercial systems, the focus is on the type of grain storage being targeted, the presence of cables in the system, moisture sensors, and whether they offer alert systems or automation. For scientific publications, this article compares the type of grain storage targeted, sensors and microcontroller used, means of data communication, interface, type of power supply, proposed machine learning analysis, and proposed consumption analysis.

Section 2 provides an overview of scientific publication in the field of grain storage and monitoring. Technological solutions for storing harvested grains are reviewed, starting with a general overview of grain storage in Section 3. Indeed, identifying and understanding the risks of storage and the quantities to be evaluated to ensure optimal storage is necessary for assessing the suitability of solutions. This section provides a context for the interest in modified atmosphere, particularly for seeds. Different modified atmosphere technologies are described in Section 5, which focus on CO_2_-enriched atmospheres. Section 5 presents the application of smart farming to grain storage, which is becoming increasingly widespread; different industrial and academic systems are also compared. Finally, section 6 gives a conclusion and development prospects.

## 2 Strategy and current research trends

**Fig. 1:**
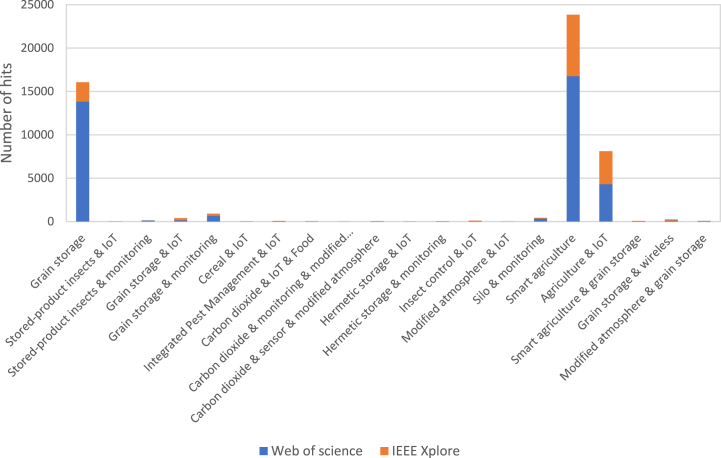
Number of results in the field of grain storage and modified atmosphere over an 8-year period (between 2016 and 2023) in Web of Science and IEEE Xplore.

This literature review was not intended to include an exhaustive search of scientific literature or be a systematic review. Instead, the objective of this review was to provide illustrative publications, works, and industry products of technological solutions for monitoring grain storage in a modified atmosphere. In this review, articles, conference proceedings, journal chapters, books, doctoral dissertations, patents, and websites were searched using keywords shown in Textbox 1, and illustrated in Fig. 1. IEEE Xplore, Web of Science, Google Scholar, Google, and Microsoft Bing were used for the search. In addition to encompassing many publishers, Google Scholar allowed the reference of patents while Google and Microsoft Bing revealed industrial products and websites. As technologies in the electronics and digital fields are rapidly evolving, focus was on the last complete six years to limit a large number of papers. Despite the best efforts, peer-reviewed papers comparing systems for monitoring artificially modified atmospheres used for grain storage were not found. Therefore, the comparison was expanded, and resulted in only a few recent papers (less than eight years old) that reviewed grain environmental monitoring systems. These brief overviews were conducted in articles that proposed a grain monitoring system for providing an upstream presentation of existing industrial solutions or previously published work. Therefore, this article provides a review of existing systems in the industry for grain environmental monitoring in a modified atmosphere and ambient air to facilitate a better understanding of the research needs of the agri-food industry in the area of monitoring systems for modified atmospheric grain storage.

Textbox 1 **Keywords used for the literature search**

• Grain storage
• Stored-product insects
• Insect control
• Grain storage
• Cereal
• Integrated Pest Management
• Food
• Agriculture
• Smart agriculture
• Modified atmosphere
• Carbon dioxide
• Sensor
• IoT
• Monitoring
• Wireless
• Hermetic storage
• Storage monitoring
• Silo monitoring

Alt-text: Textbox 1

**Fig. 2:**
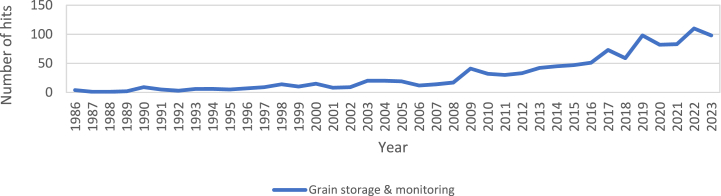
Number of occurrences of “Grain storage & monitoring” in Web of Science over the years.

Fig. 1 shows that although the term “grain storage” had more than 15,000 hits, we wanted to associate it with modified atmosphere and words representing remote connected monitoring techniques, i.e. IoT, wireless and monitoring. The “&” association between “grain storage” and the terms “IoT,” “wireless,” or “monitoring” did not exceed 1000 hits. However, although grain storage monitoring is not prevalent in the literature, it does not mean that it is not a current hot topic or can be one in the future. Fig. 2 shows that an increasing number of publications have been devoted to grain storage monitoring in recent years; this is because of the need for measurement tools and the emergence of surveillance techniques by AI.

## 3 Generalities on grain storage

### 3.1 Post-harvest

The purpose of storage is to preserve the quality of agricultural products. The post-harvest stage needs to have the technological infrastructure to support the storage of grain produced while maintaining the quality under the right conditions to prevent physical and chemical changes [9]. There are different techniques for grain storage, such as jute or polypropylene sacks, bulk storage in metal, concrete, elevated and horizontal silos, airtight storage, and storage with modified and/or controlled atmospheres. The use of temperature probes and the measurement of grain moisture content based on the hygroscopic equilibrium unit and CO2 concentration by grain mass respiration are alternatives to the monitoring of indirect qualitative variables to help monitor grain stored in bag silos, as in the following sections.

### 3.2 Main risks

Insects are a threat to crops. They can attack intact grains while the “secondary pest” species requires grain to be previously damaged (often by the first group of insects). The most common stored product insect pests of food grains are rusty grain beetle (*Cryptolestes ferrugineus* (Stephens)), red flour beetle (*Tribolium castaneum* (Herbst)), granary weevil (Sitophilus granarius (L.)), rice weevil (Sitophilus oryzae (L.)), lesser grain borer (Rhizopertha dominica (F.)), sawtoothed grain beetle (Oryzaephilus surinamensis (L.)) and Indian meal moth (Plodia interpunctella (Hubner)) [10]. Some species develop inside the grain at certain stages of their development. These hidden forms are considerably more difficult to detect and treat [7,11,12]. The main factors affecting insect development are temperature, moisture content, relative humidity, broken grains, and intergranular gas compounds (oxygen and carbon dioxide concentrations) [13]. Among these, the first two are the most important [10,14,15]. Although the optimal conditions vary slightly among insect species, the number of insects can increase rapidly at optimal temperatures between 28 and 33 °C [16]. The standard model for insect development is that of Driscoll et al. [17]. The number of insects can be determined by relative humidity, temperature, mortality temperature limiting population growth, and some coefficients; this model can then be used in decision support systems for grain storage [[18], [19]].

One-quarter of the world's food crops are contaminated with major aflatoxin [20], a toxin produced by several species of molds (fungi) [21]. Mold development is highly improbable below a certain level of water activity (usually 0.65). Mycotoxin production stops at a high water activity threshold and which differs among species [14,16,22]. The risk of mycotoxins is controlled when the grain is properly dried and stored using methods established in developed countries. However, it is still necessary to be cautious about the occurrence of excessive water activity in very localized areas of storage, especially that caused by condensation or hot spots [14]. Further, the composition of the storage atmosphere (such as high carbon dioxide concentration or extremely low oxygen concentration) can slow mold growth [23] when the water activity threshold is reached.

### 3.3 Key parameters for grain storage

The speed of grain degradation is determined by temperature and moisture. These two factors influence insect and mold growth, and thus, mycotoxins [5,16,[24], [25], [26], [27]]. Further, these two factors also impact the germination rate [25,28,29].

Temperature is a major factor that affects insect development. Insect proliferation is not possible at temperatures below 10 °C and above 45 °C. However, these thresholds can vary depending on the insect type. Further, temperature is important for germination power. The time during which the seed retains its germination capacity (in open air) is approximately halved for each 6 °C increase in the storage temperature [29]. High temperatures create stressful environmental conditions that affect grain metabolism and degrade grain quality [30].

Moisture (or water activity) is paramount to mold growth. Therefore, mold and mycotoxins are no longer problematic if the water content is low [31]. Further, moisture is an important factor in insect development [29]. Indeed, insects are no longer a threat below 9 % moisture content [14]. Three quantities need to be distinguished when moisture is discussed in the context of grain storage: Relative humidity (RH) or water activity ($a_{w}$) and grain temperature.

Different methods can be used to determine the moisture content of grains in real time such as capacitance, near-infrared, and microwave measurements [16]. The ASABE-American Society of Agricultural and Biological Engineers provides relevant information for postharvest grain management. [32], D245.6 provides data and equations on moisture relationships for agricultural products. [33]. S352.2 provides a uniform method for determining the moisture content of unground samples of agricultural seed. The information is used in crop drying calculations and in the design and analysis of food, feed and fiber storage systems. The reference laboratory method involves taking a sample of the grain and evaporating it in an oven. The initial mass and the mass measured at the out of the oven are measured (equation (1)).

(1) $$Moisture\;Content\;\left(\%\right)=\frac{m_{initial}-m_{dry}}{m_{dry}}.\;100\%$$

The moisture content of the grain can be determined from its electrical characteristics (capacitance or conductance); this technology is often used in portable moisture meters [34]. At equilibrium, the relative humidity and water activity are the same. However, relative humidity is expressed as a percentage, whereas water activity is expressed in the decimal form [35]. The equilibrium relative humidity around grains and temperature with a mathematical relationship determine the moisture content of the grains [29,36]. Most existing low-cost sensors measure relative humidity like the one proposed by Armstrong [37] or like the device developed by the Feed the Future Innovation Lab for Horticulture at UC Davis, University of California, which offers a simple, low-cost and reusable “DryCard^TM^” tool [38]. “DryCard^TM^” incorporates a cobalt chloride humidity indicator strip that changes color according to relative humidity. Within an hour, the color indicates whether the product should be used immediately or dried before storage. In general, either the water content is determined based on these equations or only the value of the relative humidity is given, and a skilled person knows how to interpret this; there are several formulas: Henderson, Chung–Pfost, Halsey, Oswin, GAB (Guggenheim, Anderson, de Boer), etc. [39]. D535 allows estimating the time needed for shelled corn with normal oil content to deteriorate to the point of losing 0.5 % of its original dry matter. Cromarty et al. (1982) developed a formula (equation (2)) to calculate the water content at equilibrium (dry weight basis) to overcome the need to determine different parameters (i.e., prior knowledge of each isotherm for each species); it is a function of temperature (T in °C), relative humidity at equilibrium ($RH_{e}$) (or water activity $a_{w}$), and oil content (fraction of dry weight $D_{0}$) .

(2) $$M_{e}=\frac{\left(1-D_{0}\right)x\;\sqrt{-440xln\left(1-a_{w}\right)}}{1.1+\frac{T}{90}}$$

As mentioned previously, the mold is no longer a major problem if the humidity remains sufficiently low (<65 %). Insect pests are the main threat to storage, as summarized by Navarro [31,40].

The conservation of germination rate is very important when grains are to be used as seeds. In Europe, seed companies are subject to standards for minimum germination rates depending on the species. Further, high germination rates are required in other cases such as malting barley, which must maintain a germination rate of 95 % [14]. Most importantly, the minimum germination rate must be ensured when storing malting barley or seeds. However, the main effects (storage time, seed MC, and storage temperature) have visible impact on seed germination and seedling emergence process. The optimal temperature for seedling growth is 20 °C, and a more comprehensive range for germination is from 20 to 35 °C. A temperature lower than the optimal range decreases the germination rate, and a higher temperature increases fungal growth. Seed size influences the quantity of water needed for germination [41].

Finally, temperature, moisture, and germination rate are good indicators of grain quality. Insects and molds are the two main risks. As a result, systems for monitoring these parameters have become indispensable for ensuring preservation quality. The following section gives a more detailed presentation of modified atmosphere grain storage technology.

## 4 Grain storage in modified atmospheres

### 4.1 Definition and principle

**Fig. 3:**
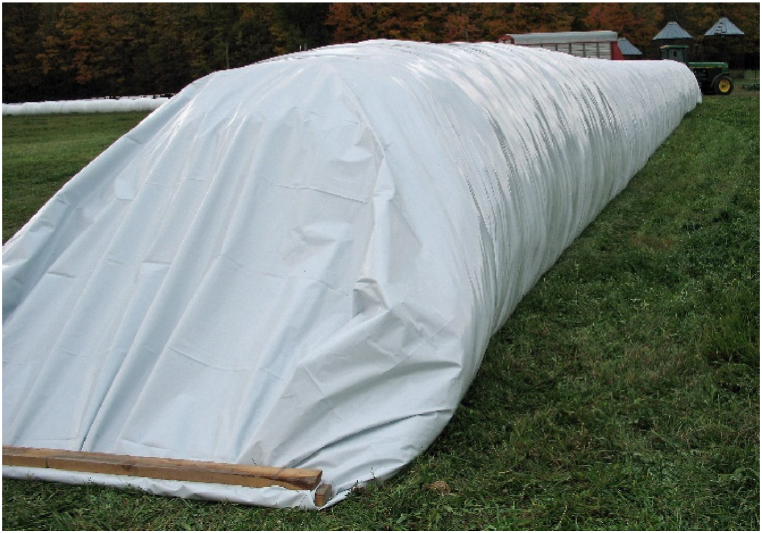
Silo bag. Source: Dale Mahalko (Creative Commons — Attribution 3.0 Unported — CC BY 3.0).

**Fig. 4:**
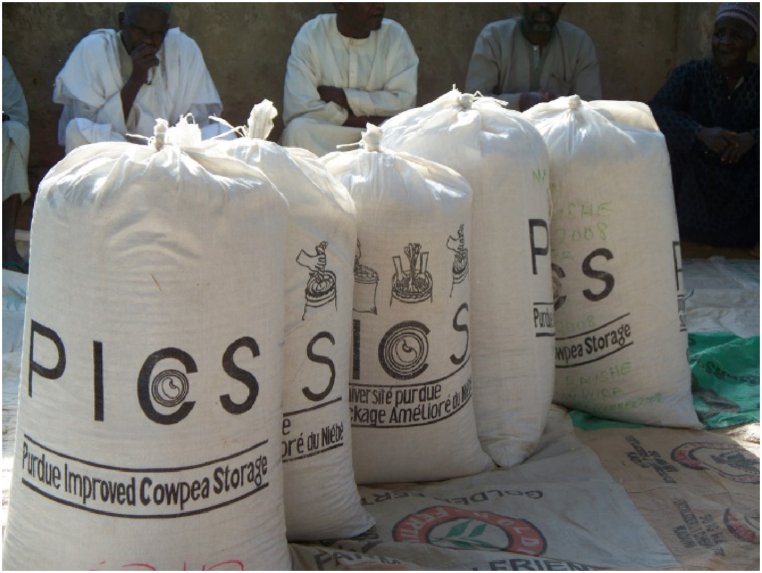
PICS bags. Source: International Institute of Tropical Agriculture (CC BY-NC-SA 2.0).

According to (Navarro,2012), the modified atmosphere is a generic term that encompasses all cases in which the composition of the atmosphere is changed by increasing the concentration of nitrogen or carbon dioxide to regulate insects and maintain product quality; this method considers low- or high-pressure atmospheres. The composition of the atmosphere can be changed biologically. Indeed, the grain, insects, and various microorganisms (which are present in the grain) respire, and therefore, consume oxygen and produce carbon dioxide. When the storage environment is well-sealed, as shown in Fig. 3, Fig. 4, an oxygen-depleted atmosphere (and slightly enriched in CO_2_) is generated. This respiration reduces the O_2_ concentration from approximately 21 % in air to 1–2 %, whereas the production of carbon dioxide increases from 0.04 % to nearly 20 % depending on the conditions [8]. Modified atmospheres not only kill insects but also have bactericidal and fungicidal effects [23,42].

### 4.2 Effectiveness on insects, molds, and germination rate

A biologically modified atmosphere inhibits the development of insects and molds, which limits deterioration [[43], [44], [45]]. The mortality of insects in a low-pressure atmosphere (partial vacuum) is attributed to hypoxia (low oxygen pressure), and mortality cannot be attributed to low pressure but rather to low oxygen partial pressure [31,46]. Navarro and Navarro [8] surmised that the oxygen level should be at least <3 % or even <1 % according to the literature for effective control of insects by injecting nitrogen. In this type of modified atmosphere, the reduction in germination rate over time is attenuated [47]. Many experiments have been conducted to determine the mortality of different insects in a CO_2_-enriched modified atmosphere [31,48] and at different stages of development depending on CO_2_ concentration and exposure time [8,[49], [50], [51], [52], [53], [54]]. Indeed, it is necessary to test these different conditions because efficiency differs based on these parameters: *“The effectiveness of Modified Atmospheres in controlling insects is dependent on various abiotic (gaseous composition, relative humidity, temperature, length of exposure, and gas pressure) and biotic (insect species, life stage, and the size and distribution of infestation) factors”* [55]. Carbon dioxide reduces the metabolism of the seed, which is an alternative for treatment against the groundnut seed borer ensuring the conservation of grain quality [30,49,55]; the modified atmosphere, especially that enriched in CO_2_ and depleted in O_2_, prevents the development of molds [22,56].

### 4.3 Examples of scale up or commercial use of CO2-enriched atmospheres

Navarro [31] reported on several installations around the world that have used a CO2-enriched atmosphere for conservation of large bulks of 10,000 to 15,000 tonnes capacity. Pons et al. [57] used hermetic bags to create a modified atmosphere very rich in CO_2_ (>75 %). The mortality of all insects (all forms, which include eggs, as emergence was tested later) was studied; these experiments were performed using rice, cocoa, and chamomile. The hermetic bags were provided by A.G. Protectpack S.L (Barcelona, Spain). Bag dimensions were 900 × 900 × 1000 cm for rice and cocoa and 900 × 900 × 1600 cm for chamomile; and were made of a polypropylene outer bag with an internal plastic bag. The results confirmed that the use of high CO2 Modified Atmosphere in gastight big bags is a feasible alternative to control the occurrence of pests, as the container did not contain any live insects at the end of the trials.

**Fig. 5:**
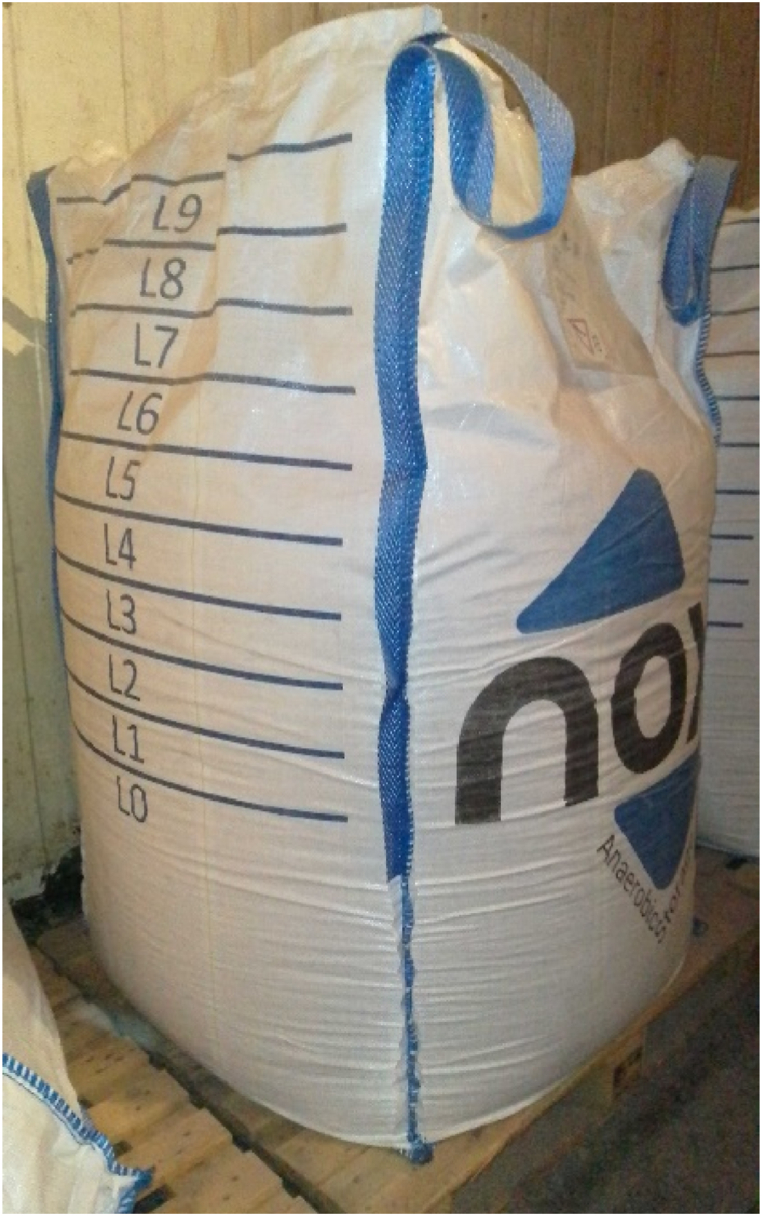
Big bag NOX.

GrainPro offers airtight bags of different sizes (as well as drying equipment) for use in developing countries. Some bags (e.g., GrainPro® Self-Verifying Cocoon™) have valves so that CO_2_ can be injected (the term used by GrainPro is “organic fumigation”). CO_2_ injection was presented in the instructional video on the product page [58,59]. VacQPack is a company that offers partial vacuum and/or modified atmospheric processing and storage with CO_2_ and/or nitrogen. A machine (VacQPack Pro) was required to create a vacuum (recommended vacuum: 50 kPa) and then inject CO_2_ and/or nitrogen. The VacQPack bags were equipped with a valve that had to be plugged with a VacQPack sticker; VacQPack offers different models such as 1.3 kg bags or a liner to wrap a big-bag that is possibly an “outer” big-bag (or a cardboard box, etc.) to protect the added liner [60,61]. Like VacQPack, vQm Packaging (2021) offers treatments and storage under partial vacuum and/or a modified atmosphere with CO_2_ and/or nitrogen. In addition, a machine (high-speed unit) was used to create a vacuum and possibly inject the gas; the vQm high-speed valve is patented. These two elements enable the expectation of a vacuum of 0.1 bar, and several vacuum- and gas-injection cycles were performed. Again, the liner (which can be of several types, including aluminum) can be protected with an exterior big bag, a cardboard box, or any other container. The French company NOX markets hermetic big bags (91 × 91 × 160 cm) composed of a very resistant woven outer bag and a hermetic inner bag (Fig. 5). They are equipped with a non-return valve system for vacuum air and another for CO_2_ injection and manual gas control. The NOX big bags were reused thrice; these bags not only eliminate insects in a single treatment but also allow storage under a modified atmosphere over time. Further, these big bags are suitable for safe transport because of their well-known size and four lifting points [62].

### 4.4 Discussion

In the context of grain storage, a modified atmosphere is enriched with CO_2_ or N_2_, and/or low or high pressure. A modified atmosphere is effective (at sufficiently high concentrations of N_2_ and CO_2_, or sufficiently low pressures) to keep grains for longer periods in storage within controlled temperature and relative humidity by eliminating all forms of insects systematically. A CO_2_-enriched atmosphere can prevent mold growth and maintain a high germination rate. The periodic reinjection of gas is required to maintain a modified N_2_-enriched atmosphere at a sufficiently high concentration. Therefore, this is restrictive for storage, although it is acceptable for a single treatment. The effectiveness of the modified atmosphere depends on gas composition, humidity, temperature, and pressure; thus, a modified atmospheric grain storage monitoring system must measure at least these quantities.

## 5 Smart agriculture applied to grain storage

### 5.1 Definition and applications of “smart agriculture”

“Smart agriculture” (or “smart farming”) is a method in which farmers can monitor their fields and activities remotely; this helps limit human actions [63]. Precision agriculture focuses on optimization [64]. According to Alonso et al. [65], “smart farming” uses information and data technologies to provide a more comprehensive analysis than precision farming, which considers location, history, real-time data, and predictions before taking action. These terms result in the use of the Internet of Things (IoT) technology (e.g., sensor network, wireless communication, embedded data processing) [66]. The subject of “smart agriculture” and “precision agriculture” has received attention from many researchers. A large number of studies have been published in recent years [[63], [67], [68]]; they provided a fairly comprehensive overview of Internet of Things in smart farming, which includes a systematic review of publications from 2011 to 2019 [63]. There are different uses and applications for smart agriculture: agricultural robots, GPS tracking and location, RF Identification, connected weather stations (frost prevention, etc.), automated irrigation (and fertilizers) (optimization), disease detection with artificial intelligence from images, greenhouse automation, and harvest monitoring. However, this article focuses on automatic monitoring grains during storage. The monitoring and control of grain storage provides more relevant information in real time, and enables us to predict any changes in parameters that could jeopardize the stored contents.

### 5.2 Commercial applications for grain-storage monitoring

#### 5.2.1 Traditional thermometric solutions

The elements mentioned in this section are not directly related to the definition of “smart agriculture,” mentioned above. However, a more global view of grain monitoring is required to understand the contribution of “smart agriculture” to grain storage.

A widespread practice now (used for more than 60 years) is to place cables with temperature sensors (thermocouples or thermistors) at regular intervals in the grain (stored in silos or concrete granaries) [34]. These cables are attached to the top of the structure before being filled with grain. They are used to monitor temperature and its potential evolution, in order to trigger ventilation or disinsectisation in the event of a rise in temperature inside the silo. Examples include AGI CMC [69], [70], Isoelectric [71], [72], [73], Tornum [74], Inteqnion [75], Pfeuffer [76], etc. These solutions display the data directly on a screen.

#### 5.2.2 Connected sensors systems

Some companies offer connected systems that measure the temperature and humidity in the silo. This information can be displayed on user interfaces (web pages, applications) or used to generate alerts in the case of anomalies. Further, there are solutions for the automatic control of ventilation. For example, the French company [77] optimized the use of ventilation based on temperature fluctuations. Some companies offered even more completely connected solutions for the silo. Moreover, CO_2_ measurements are sometimes performed to detect the presence of insects or the development of molds, as proposed by Amber Ag [78]. AgroLog [79] and Eye-Grain [80] offered a fire detector. Most existing solutions are limited to storage in silos (or concrete attics), and a few companies are beginning to offer solutions for other types of storage. TeleSense® [81] sells wireless sensors inserted into the grain that measures temperature and relative humidity and alerts the user when pre-set threshold values are exceeded. Centaur analytics offers a generic sensor that can be adapted to different storage forms (silo, silo bag and metal shipping containers); it measures temperature, humidity, and CO_2_ (as in the solutions presented above), and it monitors the concentration of phosphine during fumigations. An analysis has been proposed to guarantee insect mortality during fumigation (with phosphine) [82,83]. Storage in silo bags (hermetically sealed cylindrical bags up to a few hundred meters long in the field) is widespread in North and South American countries, especially Argentina. Companies offer probes to be inserted at regular distances in the silo bag to automatically measure the temperature, humidity, and even CO_2_ to detect mold development. Some of these devices provide an alarm in the case of vandalism [[84], [85], [86], [87], [88]]. The system presented by Bartosik et al. [89] requires the intervention of the operator; however, it allows data collection at a lower cost: the measurement of the CO_2_ concentration of silo bags with Silcheck [90]. An operator is required to move the measuring device and identify the bag using an RFID tag. Thus, this is only a semi-automatic measurement system and is not listed in Table 1. Villers et al. [91] introduced GrainPro EcoWiSe™ to monitor the temperature, humidity, oxygen, and carbon dioxide levels in a GrainPro Cocoon. Located in a sealed bag, it communicates wirelessly with a computer located up to 500 m away (or even further with a repeater). The battery has a life span of 5 years; this product is available for sale on the GrainPro website only from mid-2022 but the device monitors only the temperature, humidity, and carbon dioxide (up to 80 %) and not oxygen. It was previously proposed as an optional add-on to the GrainPro self-verifying cocoon [59].

**Table 1:** Comparison of commercial connected sensor systems.

Brand Company	Cables	Humidity sensor	Other sensors	Alert/Automation	Grain storage (in natural air)	Reference
GRAINX	Yes	Yes	–	● Alert	Silo	GrainX [92]
*BinCheck Deluxe* IntelliFarms	Yes	Yes	–	●Alert	Bin storage	IntelliFarms Northern Division, Inc. [93]
*DuoLine STAR mobile PLUS & cables* Pfeuffer	Yes	(Optional external humidity sensor)	–	●(optional) Alert	Silo	Pfeuffer GmbH [94]
Chang Yu Technology Corp. Ltd. (CYTC)	Yes	Depending on the version	–	Depending on the version: ●Alert ●Automatic ventilation	Silo	CYTC [95]
*SAFETRACK wireless grain monitor system* SafeGrain Inc.	Yes	No	–	●Alert ●(optional) Automatic ventilation	Bin	Safe-Grain, Inc. [96]
*BIN-SENSE Live®* IntraGrain Technologies	Yes	Yes	–	●Alert ●Automatic ventilation	Silo (flat bottom, hopper bottom and elevators)	IntraGrain Technologies [97]
ERGSON®	Yes	Yes	–	●Alert ●Automatic ventilation	Silo	Ergson GmbH [98]
GESCASER	Yes	Yes	●Anti-condensation probe ●Weather station	●Alert ●Automatic ventilation	Silo	Gescaser [99]
*BinManager* IntelliFarms	Yes	Yes		●Alert ●Fan & heater operation	Bin storage	IntelliFarms Northern Division [100]
*OPI Blue* OPI™	Yes	Yes	–	●Alert ●Fan control	Silo	OPI systems Inc [101]
*DuoLine STAR mobile PLUS & Wireless measuring probes* Pfeuffer	Probe	(Optional external humidity sensor)	–	●(optional) Alert	Flat storage (until 10 m depth)	Pfeuffer GmbH [102]
*U-sensor Plus* CartaSense	No	Yes	–	–	Not specific	Cartasense [103]
*Ball* Telesense	No	Depending on the version	–	●Alert	Not specific	Telesense [104]
*THERMOPOINT* NIVELCO	Probe (<30 m)	No	–	●Alert	Flat storage (until 30 m depth)	Semrad [105]
*Tango M probe* Quanturi	(2/3/4 m)-probe	No	–	●Alert	Flat storage	Quanturi [106]
Javelot	2 m-probe or cables	No	–	●Alert ●Automatic ventilation	Flat storage or vertical storage	Javelot [77]
*BULLSEYE BIN CONTROLLER (CO*_*2*_*sensor & ADLink)* GSI	Yes	Yes	●CO_2_ sensor and temperature & humidity sensors headspace ●Static pressure	●Alert	Bin storage	GSI [107]
*AgroLog TMS6000* AgroLog	Yes	Yes	●CO_2_ sensor and temperature & humidity sensors headspace ●Anti-condensation probe ●Weather station ●Level sensor	●Alert ●Automatic ventilation ●Open system to access advanced computation	Silo	AgroLog [79]
*ACE AIR* amber	No	Headspace only	●CO_2_ sensor	●Alert ●Automatic ventilation	Bin	Amber Agriculture, Inc. [78]
*Spider* Telesense	No	Headspace and bottom space only	●CO_2_ sensor	–	Ventilated Silo	TeleSense [104]
*iGrain* Eye-Grain	Yes	Yes	●CO_2_ sensor ●Humidity sensor headspace ●Fire sensor (CO and CO_2_) ●Plenum Pressure sensor ●Weather station ●Radar level sensor	●Alert (even spoilage detection) ●Automatic ventilation	Silo	Eye-Grain [80]
*Spear* or *CellSpear* Telesense	Probe	Yes	●Location monitoring	●Alert	Flat Storage/Warehouses/Barges/Railcars	TeleSense [104]
Centaur Analytics Inc.	No	Yes	●Phosphine ●CO_2_ sensor ●Volume Sensing	●Alert ●Automatic ventilation ●CFD Modelization ●Insect mortality during phosphine treatment	Silo/Silo bag (even with fumigation or heat treatment)	Bantas et al. [83] CENTAUR ANALYTICS, INC [19]
SensDRB GmbH	No	Yes (capacitive moisture content and air humidity sensor)	●CO_2_ sensor (Explor-IR M) ●O_2_ sensor (ME2-O2) ●accelerometer	●Alert (biological activity or theft)	Silo bag	Barrettino et al. [84,108] SensDRB GmbH [87]
LESS Industries	No	Yes	●CO_2_ sensor	●Alert (biological activity)	Silo bag	LESS Industries [86]
DekaGB	No	Yes (capacitive moisture content and air humidity sensor)	●CO_2_ sensor ●Outdoor temperature and humidity	●Alert (biological activity or sensor removal)	Silo bag	DekaGB [85]
*Smart Silo bag* or *Smart Barge* Wiagro	No	Yes (air humidity sensor)	●CO_2_ sensor (Explor-IR) ●GPS ●Accelerometer	●Alert	Silo bag or Barge (Depending on the device)	Wiagro [88]
*Ecowise* GrainPro	No	Yes	●CO_2_ sensor (up to 80 %)	●Alert	GrainPro Cocoon (hermetic bag)	GrainPro [109] Ocreto et al. [110] Villers et al. [91]

#### 5.2.3 Electromagnetic imaging

Electromagnetic imaging (MRI) is widely used in the medical field. The differences in the dielectric properties of the materials are detected. An array of electromagnetic antennas creates an image of grain storage as a function of dielectric properties (which vary with humidity and temperature) as detailed in a review by LoVetri et al. [111]. Therefore, it is possible to determine the moisture content of the grains in the silo (and in 3D); this can be realized using GrainViz [112]: This technology is efficient, but expensive [34].

#### 5.2.4 Insect detection systems: Acoustic probes and connected traps

The detection of noise produced by insects has been studied for more than 35 years [18]. Different methods have been proposed by acoustic devices [113]. The French company Systelia (www.systelia.fr) markets different acoustic probes depending on their use (long-term storage, portable version, etc.). Acoustic emission consulting was conducted by an American company that produced the probes. The AED-2000L and 2010 L acoustic probes cost 200 USD each, and the production was discontinued in 2019 [114].

Insect traps are “plastic tubes” inserted on top of the grain to capture insects to track their development and the species present [18]; this makes it possible to adapt it to disinfection. However, traditional traps must be inspected regularly, and it can lead to accidents in silos [34]. Connected traps exist today, and this allows the user to count insects that fall into the trap. Insects are attracted by pheromones, and an infrared barrier counts every insect that falls into the trap. Some traps even identify the type of species with the size of the insects such as the Insector from OPI Systems Inc. (https://www.grainstoragesolutions.ca/opi-systems/).

### 5.3 Experimental devices

Advancements in monitoring technology have enabled greater quality control of stored products through indirect parameter measurements, which help predict quantitative and qualitative losses and inform decision-making. This section presents an overview of monitoring techniques that have been developed, and in some cases deployed in real-life conditions, and published in the scientific literature but not yet on the market. They are based on a combination of sensors, often combined with data analysis techniques recently derived from AI. Among application cases, we will focus a little more on the monitoring of modified atmosphere storage silos presented in section 4.

#### 5.3.1 Monitoring systems based on CO_2_ sensors

Many studies have proposed measuring CO_2_ concentration for insect or mold detection, but few have proposed a system for continuous monitoring CO_2_ concentration in an atmosphere artificially enriched with CO_2_.

The study by Gonzales et al. [115] is one of the first studies published on the use of relative humidity, temperature, and carbon dioxide sensors for grain monitoring. They used six nondispersive infrared CO_2_ sensors (Ventostat 8102), six temperature and humidity sensors (SHT75), and one airflow sensor (FL-806 Ω) to evaluate their potential to predict adverse storage conditions of the wheat such as a hot spot with high moisture (>14 %).

Ubhi and Sadaka [116] determined that it was possible to estimate the rate of respiration by using a pressure sensor and developing a correlation between the pressure drop associated with the heat of respiration and CO2 molecular concentration using gas equation.

Bettahar [18] used CO2 concentrations to detect insect development as part of integrated pest management using a probe integrated many sensors (temperature, humidity, CO2, acoustic).

Sindwani et al., Kaushik and Singhai [117,118] proposed a system to detect mold contamination of grain.

The system proposed by Tedla et al. [119] was intended to be comprehensive: an infrared motion detector (to detect rodents, e.g., a CO_2_ sensor to detect abnormal activity (insects or mold), temperature, humidity (DHT22), and an automated drying, cooling, or controlled light system. An SMS is sent in the case of an alert and a web interface is used to obtain the information. For the design of this system, the robustness of the chosen elements was verified for adaptation to the sub-Saharan climate.

Kodali et al. [120] proposed an experimental device that monitors the temperature, humidity (DHT11), and presence of CO_2_ (MQ135); their device helped in the management of the warehouse stock by the principle of “first in, first out.” The expiration dates were considered and data were visualized using Amazon QuickSight. This system used Amazon Web's push bullet service to send alerts. Autonomy was not discussed in this study, and a GSM GPRS module was provided when the grain was transported.

Devi et al. [121] proposed a system that monitors temperature and humidity (DHT11), CO_2_ concentration (up to 5000 ppm with a MG-811 sensor) to detect grain contamination, and a presence sensor (PIR). When the PIR sensor detects a rodent, an ultra-sonic deterrent is activated. Ventilation and light are activated depending on temperature and humidity levels. They did not explain if the triggering logic is a threshold or if it's a more advanced logic. Unfortunately, this prototype has not been tested with stored grain.

Hema et al. [122] proposed an efficient system that comprises a temperature and relative humidity sensor, light-dependent resistor, and CO_2_ sensors inside grain storage containers. Although the CO_2_ concentration was measured, the value was simply displayed, and no analysis was performed to detect insects or molds. The system was turned on the fan when the temperature reached a threshold to reduce it; the system turned on the humidifier when the humidity exceeded a threshold level. However, the system did not perform well when the humidity was too high for safe storage; this is more common in practice (especially because of the respiration process). Although the system was tested in the laboratory using a scale model, there was no testing using full-size grain container.

Kumar et al. [26] proposed a multi-sensor monitoring device in an hermetic partial vacuum storage. They found that the frequent opening of a sealed container for manual monitoring disrupts the modified environment as it was also reported by Tubbs et al. [123]. The monitored variables were temperature and humidity (DHT22), pressure (BMP-180), and a CO_2_ sensor (COZIR, with a measurement range of 0–100 %). An alert was activated (with a buzzer) when the partial vacuum was no longer sufficient to maintain the pressure below 300 mmHg; the data were not sent wirelessly but by wire to a display and storage device. It used an Arduino Mega 2560 microcontroller with an LCD display, LEDs, and a few physical buttons for the interface. The system was supplied with alternating current (AC) power (220 V, 50 Hz). In the aforementioned systems, CO_2_ data were used to detect the phenomenon of respiration (and thus, the presence of insects and molds). CO_2_ sensors generally have very limited amplitude (often a maximum of 0–5%, which is the most common for monitoring ambient air). However, it is necessary to use CO_2_ sensors to measure concentrations between 40 % and 100 % to monitor an artificially enriched CO_2_ atmosphere. CO2 concentration above 60 % by volume at a relatively constant RH has been proven to be a more environmental-friendly fumigant.

Zou et al. [124] tested the sensor system in a controlled atmosphere for temperature, water, and CO2; the CO2 sensor used was with a Telaire 6004, which has a measurement range of 0–2000 ppm or approximately 0–2000 mg/m3 as specified in their study.

Numerous studies exist on the use of sensors for monitoring ambient grain parameters in different containers, but no experimental solution has been tested in hermetic storage enriched with CO2 and for a long period of time. The articles by [9] and [125] synthesize current studies that address the use of low-cost sensors, Internet of Things (IoT) precepts and Machine Learning (ML) technologies in post-harvest grain quality monitoring. On this basis, we believe it is possible that a combination of these different technologies could meet most monitoring needs in any type of storage environment.

The authors of this review paper are working on a smart multisensor embedded device for continuous measurement of parameters of a hermetic CO2-enriched big bag storage: temperature, humidity, pressure, accelerometry, CO2, combined with a web interface for monitoring the evolution or change of these parameters. An article is currently in progress.

#### 5.3.2 Grain environment monitoring devices associated with artificial intelligence

Most commercial and experimental systems use only a threshold condition to perform actions such as activating ventilation or generating an alarm. This section focus on systems with advanced decision logic that use learning methods are presented.

Some studies have proposed the prediction of temperature as one of the most important factors in grain storage in ambient air [5,[126], [127], [128], [129], [130]] or humidity, which is another important factor [131]. Bettahar [18] used machine learning in their temperature prediction model (as well as learning methods for the acoustic classification to detect insects). Therefore, some studies have focused on predicting this parameter. Sindwani et al. [117] used machine-learning algorithms to predict the relative humidity for the next five days using relative humidity data collected at a particular temperature; a comparison of several machine-learning algorithms is presented in Table 2. Ahmad et al. [132] proposed an alternative approach to paddy drying using hot air to maintain moisture content within controlled values based on historical patterns and internal and external environmental parameters. Machine learning models were trained using supervised learning techniques on data obtained from Internet of Things (IoT)-enabled smart silos. Some studies used machine-learning methods to directly determine the grain quality and whether storage is at risk [118,133,134]. Cui et al. [135] used a back propagation neural network to classify the grain conditions, including inventory changes and routine operations (aeration) based on the temperature contour map of the stored grain. Kaushik and Singhai [136] compared several machine learning algorithms on their ability to predict the quality of stored grain based on temperature, moisture content, and CO2 concentration. Random Forest machine learning algorithm performed better in classifying grain based on experimental results.

**Table 2:** Overview table of multisensor environmental monitoring systems between 2016 and 2020 (over five years) “X”: With information present in the publication, these data cannot be provided.

Reference	Grain storage	Purpose of publication	Sensors & Microcontroller & Actuator	Wireless data communication	Learning method	User Interface	Supply & Power Consumption Analysis
Ahmad et al. [132]	Silo-bin	Presented a methodology to predict paddy moisture content based on historical patterns, and internal and external environmental parameters.	7 Unknown temperature and relative humidity sensors 2 Unknown airflow sensors	X	Yes: Use of learning method to predict moisture content during drying.	X	No
Akila and Shalini [137]	X	Proposed a storage management system to monitor the quality of food grains that measure ammonia gas.	1 HS1101 (Humidity capacitive sensor) 1 Transcat 7010T (Resistance temperature detector) 1 MQ135 (Ammonia gas sensor) 3 Arduino Due R3 (MCU) for each sensor	Wi-Fi	No (Only threshold alert)	X	No consumption analysis
Banerjee et al. [138]	Storage warehouse	Developed an IoT instrumented warehouse system to have live data at a very low cost.	1 DHT22 (Temperature & RH) 1 HC-SR501 (Motion sensor) 1 KY-002 (Shock sensor) 1 fire sensor (Detects bright light source) 1 MQ135 (CO sensor to detect fire) 1 ESP32 (MCU)	Wi-Fi (MQTT protocol)	No (Only fire alert)	Node-red dashboard SMS & email every 30 min	No consumption analysis
Bettahar [18]	Silo	Presented SILOCARE, an innovation system with “a predictive model of the infestation development, which comprises coupling a thermal model of the silo with a biological model describing the insect's growth.” This system is composed of: ●A detection system at the entrance of silos. ●A supervision of the top of the hottest heap of grain. ●A monitoring of the heap of grain in its entire height. “These developments are implementing multiple detection modes (temperature, humidity, acoustic probe, and CO_2_).”	The detection system at the entrance (SILOTEST 2): 2 MULTICOMP ABT-441-RC (piezoelectric sensor) 1 SHT21 (Temperature sensor) 1 Fubarino SD (Microcontroller) Electronic probe (SILOTEST 1): 1 Unknown acoustic sensor (Microphone) 1 Unknown acoustic piezo sensor 2 SHT21 (Temperature & RH sensor) 1 Infrared counter for insects counting 1 Custom controller (With PIC16F877) Cables (SILOTEST 3), for each module (every 3 m): 1 COZIR GC-0013 (CO_2_ sensor) 1 SHT75 (Temperature and humidity sensor) 1 MULTICOMP MCABT-455-RC (Piezoelectric sensor) 1 PIC 18 F (Microcontroller)	No: SILOTEST2: USB SILOTEST1: RS232 SILOTEST3: RS232 and Ethernet (TCP-IP)	Yes: A thermal predictive model with learning and learning method for acoustic classification.	Local computer software (SILOSOFT made in Java and with MySQL)	SILOTEST1&2&3: mains supply No consumption analysis
Dal-uyen et al. [139]	Small silo	Developed a system to ensure safe storage by continuously monitoring temperature and relative humidity, and by measuring moisture content nondestructively.	7 SHT21 (Temperature & RH) 1 Arduino Mega 2560 1 Heater and fan	GSM (SMS)	No	Data sent by SMS	No consumption analysis
Devi et al. [121]	Warehouse	Presented a monitoring system to control temperature and humidity and monitor rodents and mold apparition.	3 DHT11 (Temperature & RH) 1 MG-811 (Carbon dioxide sensor) 1 Passive Infrared sensor (Motion sensor) 1 Arduino Uno (MCU) 1 ESP8266 Wi-Fi (MCU for Wireless connection) 1 Ultrasonic repellent device 1 Fan 1 Light	Wi-Fi	No	Application (Blynk) with notification	No consumption analysis
Furtado et al. [140]	Silo	Developed *“a low-cost automated drying system and tested its effectiveness in the drying process of cocoa beans through a case study.”*	5 DHT22 (Temperature & RH) 1 Esp8266-12E microcontroller 1 Relay for fan operations	X	No (Fan is only controlled by user's action).	Application for android smartphone: Data Silo	Main Supply (110 V AC) No consumption analysis
Hema et al. [122]	Bin	Proposed an effective system composed of sensors inside grain storage containers.	1 DHT11 (Temperature & RH) 1 Light dependent resistor 1 Unknown CO_2_ sensor 1 ATMEL Atmega328P controller 1 ESP8266 Wi-Fi 1 Exhaust fan 1 Humidifier 1 Alert	Wi-Fi	No (Fan and/or humidifier: threshold activation)	ThingSpeak webpage (not user-friendly)	No consumption analysis
Kanaan and Bavkara [133]	Silo bag	Presented a wireless sensor network (without resource intensive monitoring like done with Silcheck [141]) to predict the condition of silobag conditions using machine learning techniques and compared them.	Each slave node: Several SHT10 (Temperature and humidity sensor) Master node: 1 Raspberry Pi 3 (Master node)	Wi-Fi & 3G	Yes: Artificial neural networks algorithm to classify if the storage is safe, risky, or dangerous.	A MATLAB-based GUI (Graphical User Interface)	No consumption analysis
Kaushik and Singhai [118]	(Metallic) bin	Presented the design of an upcoming real time and integrated sensing system that monitors environmental factors for the early detection of contamination in stored grain.	X Unknown CO_2_ sensors X Unknown O_2_ sensors X Unknown temperature and humidity sensors 1 Arduino Mega 2560 1 FPGA (Field Programmable Gate Arrays)	Wi-Fi	Yes (upcoming: learning methods for classification between “no spoilage,” “early spoilage,” “severe spoilage,” and “early insect infestation”)	X	No consumption analysis
Kaushik and Singhai [136]	Granary	Proposed a system to monitor temperature, humidity, CO_2_ concentration. Several machine learning algorithms are compared on their ability to predict the quality of stored grain.	1 SHT11 (Temperature & RH) 1 Senseair K30 (Carbon dioxide sensor) 1 RF module 1 FPGA 1 ATMega2560 (MCU)	434 MHz	Yes, machine learning algorithms: K-Nearest Neighbor, Random Forest, and Linear Regression	X	No consumption analysis
Kodali et al. [120]	Warehouse and during transport	Presented a system that consists of a microcontroller and various sensors (temperature, humidity, and CO_2_), and it also helps the “First In, First Out” logistic and with expiration dates.	Each slave node: 1 DHT11 (Temperature & RH) 1 MQ-135 (Carbon dioxide sensor) 1 ESP8266 Wi-Fi (MCU)	Wi-Fi (& GSM GPRS module when grains are in transport)	No (Threshold for temperature and humidity)	Amazon Web Services Amazon Quicksight (graph only for temperature and humidity). Alerts via pushbullet service.	No consumption analysis
Kumar et al. [26]	Vacuum hermetic (200–400 mmHg)	Compared hermetic-vacuum storage with conventional phosphine fumigated storage (for 6 months).	4 DHT-22 (Temperature & RH): 3 inside and 1 outside 1 BMP-180 (Pressure) 1 GC-0016 aka Explor IR W 100 (CO_2_ sensor 0–100 %) 1 Arduino Mega 2560 (MCU) 1 Piezoelectric buzzer	No (Data displayed directly on an LCD screen and stored on an SD card)	No (Only threshold alert)	LCD display, LED, user buttons. Buzzer alert.	Powered by mains supply No consumption analysis
Li et al. [142]	Concrete silo	Presented a smart cooling-aeration system to optimize the control of aeration fans.	Headspace: 1 Unknown humidity and temperature sensor Grain space: 4 Temperature cables (with 11 DS1820 in each) No information about the microcontroller Centrifugal fan	No	No (but elaborated models to determine the moisture content and aeration windows)	X	No consumption analysis for the monitoring system but for the cooling/aeration
Martyn et al. [143]	Silo/Bin	Presented QCONTROL: A wireless system for temperature and humidity monitoring to reduce the cost of cable thermometers installation and operation.	For each standalone temperature/humidity sensor: 1 Unknown temperature sensor 1 Unknown humidity sensor For each rod: 3 Unknown temperature sensors 1 (optional) Relay for ventilation	433.92 MHz (TCP/IP)	No	X	Lithium battery No consumption analysis
Muller-Blenkle et al. [113]	Silo grain	Presented a "Beetle Sound Tube" system as an acoustic early detection system for insects in grain.	1 microphone 3 climate sensors (EE 071, E + E Elektronik, Engerwitzdorf, Aus-tria) to record temperature and relative humidity. 1 microcontroller (RPi).	LAN ethernet, LTE modem, WLAN router	Not yet but discussed	X	No consumption analysis
Mihajlo et al. [144]	Silo grain	Described the *“design of HDS Tara system, an automated system for stored grain monitoring based on the NB-IoT communication technology.”*	Housing (on the ceiling): 1 MB7040 I2CXL-MaxSonar-WR ultrasonic sensor (distance sensor to monitor the grain level) 1 Sodaq SARA AFF R410M board with ATSAMD21J18 MCU Probe (up to 10 m): 4 HDC1080 (Temperature, relative humidity, and moisture sensors)	NB-IoT (MQTT)	No currently (threshold): In further improvements, they proposed to determine the safe storage time before the grain starts to spoil.	Cumulocity IoT platform: Web application (user-friendly) SMS and e-mail alert	A simple Li-Ion battery No consumption analysis
Onibonoje and Olowu [145]	X	Presented *“the use of a wireless sensor network to monitor in real-time the variation in temperature, humidity and light illumination parameters […] and automatically initializing control measure for the effect whenever necessary.”*	Each node: 1 GL5528 (Light sensor) 1 DHT22 (Temperature & RH) 1 Arduino Pro mini 1 Relay for fan operation	Zigbee Xbee module (for nodes) Serial port between gateway and computer	X	PC monitor: Any information about interface/software.	9 V alkaline battery No consumption analysis
Onibonoje et al. [146]	Silo	Designed and developed a low-power and low-cost wireless monitoring system for storing bulk grains.	For each node: 1 DHT22 (Temperature & RH) 1 Grove-LDR GL5528 (Light sensor) 1 Arduino Pro (MCU)	Node-coordinator: Xbee - Zigbee (Mesh)	No (Only threshold alert)	LabVIEW (Not user friendly)	9 V alkaline (1200 mAh) Consumption analysis Optimization of the Arduino consumption, etc.
Parvin et al. [147]	Silo	Proposed models for monitoring grain storage optimized in the number and positioning of nodes.	Only examples of sensors: DS18B20 (temperature sensor), SHT75 (temperature and humidity sensor), or SDP610 (pressure sensor)	X	No (Threshold logic)	X	Determination of energy consumption by clusterhead
Román and Hensel [148]	Small storage (Ethiopian drying and storage structure)	Presented a humidistat to control a solar-powered DC fan for small storage in remote areas; it is optimized in cost.	1 DHT22 (humidity sensor) 1 Microcontroller board Pro Mini 1 Latching relay HFD2-005-S-L2	No	No (threshold)	No user interface.	Humidistat supplied by four AA batteries. Consumption analysis
Sheng et al. [149]	Bulk grain silos	Presented a comprehensive monitoring-control system to manage several silos (with belt conveyors, etc.)	X No accurate information about them just the presence, “93 Analog input points, 562 Digital input points,” as X Unknown temperature sensors of explosion venting ports X Current sensors for belt conveyors, buckets elevators, fans X Hopper weighers “89 digital output points” as X Buzzer X Fans X Elevators and conveyors 1 S S7-300PLC (Programmable Logic Controller) with ET200M (I/O module)	No: PROFIBUS field bus	X	2 PC monitors and one engineer station (with WINCC)	Mains supply No consumption analysis
Sindwani et al. [117]	Large depots	Proposed a system to monitor temperature, humidity, and carbon dioxide concentration, and compared different methods to predict the relative humidity at a particular temperature.	Each node: 1 DHT22 (Temperature & RH) 1 CDM7160 (Carbon dioxide) 1 ESP8266 Wi-Fi (MCU)	Wi-Fi	Yes: ●RH predictions for the next five days (comparison of different methods) ●Calculated “loss percentage” (IF-ELSE conditions)	Free web application herokuapp.com	2200 mAh Lithium-ion rechargeable batteries Consumption analysis
Srilakshmipathy et al. [150]	Silos	Presented a system with “*transmitter node and receiver node, transmitter node includes an Arduino promini microcontroller unit, a LoRa RA02 module, and a BME280 sensor that measures temperature, humidity and transmits both the values to the receiver node with the help of LoRa.”*	1 BME280 (Temperature & RH) 1 Lora RA02 module (RF Module) 1 Arduino ProMini (MCU)	LoRa	No	Dashboard (with Node-Red)	AAA batteries Consumption of 0.045W
Tedla et al. [119]	Storage house (in Sub-Saharan region)	Presented a complete automation system for grain storage houses designed to resist the Sub-Saharan climate.	For each node: 1 DHT22 (Temperature & RH) 1 Passive Infrared sensor (Motion sensor) 1 ESP8266 Wi-Fi (MCU) Gateway/web server: 1 MH-Z19 (CO_2_ gas sensor) 1 Raspberry Pi Drying Fan Cooling Fan Illumination	Wi-Fi (MQTT protocol)	No (Only threshold alert)	Webpage (with or without internet locally) SMS for alerts	No consumption analysis
Wang et al. [151]	“*Special equipment storage and transportation*”	Presented “*a wireless real-time status monitoring system […] based on ZigBee and introduced the hardware and software structure of the system. It proved that the system can accurately monitor the temperature and humidity, acceleration, and pressure*”	For sensor node: 1 SHT11 (Temperature & RH) 1 BMP085 (Pressure sensor) 1 LIS302DL (Accelerometer) 1 Unknown microcontroller Coordinator node: 1 Board with a STM32F4X (MCU) 1 OLED screen 1 Keyboard	Zigbee	No	OLED screen and user buttons.	No consumption analysis
Wu et al. [152]	Dryer storage	Presented a system that can successfully dry corn with an accumulated temperature control model used with monitoring data.	12 Unknown grain temperature sensors 3 Unknown hot air temperature sensors 12 Unknown ambient temperature & RH sensors 12 Unknown exhaust gas temperature & RH sensors 2 Unknown level sensors 1 Laser level sensor 1 Programmable Logic Controller (PLC) X “And other hardware peripheral circuit” notably dryer	No: RS232/RS485	No (However, the accumulated temperature model for the drying operation instead of the traditional threshold method).	LabVIEW on a PC monitor	Mains supply No consumption analysis
Xiaodong et al. [153]	Granary	Presented a system with an embedded operating system to monitor environmental parameters such as the grain temperature, humidity, and video image of the granary.	1 SHT10 (Temperature & RH sensor) 1 Unknown camera 1 S3C641OX (Microcontroller)	Ethernet communication (or GPRS temporary if Ethernet fails)	No	LCD graphical user interface	No consumption analysis
Zhao et al. [154]	Large granary	Presented a temperature and humidity monitoring system based on optical fiber sensors system and associated experiment in a 2700-ton wheat granary.	Optical fiber (with 8 sensors elements in the test: Temperature and humidity) 1 Splitter 1 Coupler 1 Light source 1 Fiber Bragg Grating wavelength interrogation unit 1 Computer	No (optical fiber and wire)	No	Computer	No consumption analysis
Zhang and Zhang [155]	Large-scale granaries	Proposed a system to monitor environmental parameters such as temperature, humidity, and grain reserves.	Each node: 1 DHT11 (Temperature & RH) 1 Unknown pressor sensor 1 STM32F103C8T6 (Microcontroller) LED alarm	CAN and GSM/GPRS	No (Threshold logic to alert).	Computer software	No consumption analysis
Zou et al. [124]	Granary	Proposed a system based on the ZigBee network and ARM controller to monitor temperature, humidity, CO_2_ concentration, and grain water content. A fuzzy intelligent algorithm is implemented in the ARM controller.	Acquisition node: 1 AD590 (Temperature sensor: environment and grain) 1 SHT75 (Environmental humidity) 1 T6004 (Carbon dioxide) 1 TDC220 (Grain water content) Control node: Several unknown controls units ARM controller: 1 S3C6410 (MCU)	Zigbee (for nodes) Wi-Fi (between controller and PC monitor)	Yes (Fuzzy logic)	7″ LCD touch screen PC monitor	No consumption analysis

#### 5.3.3 Systems using electromagnetic waves for grain monitoring

Asefi et al. [156] investigated the use of radiofrequency imaging inside silos to determine anomalies such as areas with higher water content; this technique is called “RF grain bin imaging (RFGBI).” Indeed, there is considerable research on the electrical properties of grains as a function of temperature or water content. Further, Ershov et al. [157] proposed the automated determination of the moisture content of grains stored on the ground in concrete granaries using electromagnetic wave measurements. The system was located on the floor of a concrete granary and a message was sent to the operator if the humidity threshold was reached.

#### 5.3.4 Systems without CO_2_ sensors, electromagnetic imaging, or machine-learning methods

Akila and Shalini [137] proposed a prototype to identify the quality of food grains using sensors such as temperature, humidity and ammonia gas. Ammonia is a colorless, pungent gas produced by fungi that decompose grains. Onibonoje et al. [146], described a mesh network sensor system using Zigbee/Xbee wireless technology, and the temperature, humidity, and light intensity were recorded. Although the project was only performed using Arduino Pro mini, an effort was made to reduce the consumption of the active phases estimated in a paper published in 2019. The graphical user interface was very limited although it is simple and efficient (LabVIEW). Trancă et al. [158] presented a ZigBee-based solution (SiloSense) using a network of custom-made boards to monitor silo-grain conditions. However, only the temperature was monitored using six thermocouples (temperature sensors) in each connected cable; they determined the power consumption and offered a 12 V/1000 mAh battery and 1 W/12 V solar panel. Wang et al. [151] presented a wireless monitoring system for “special equipment storage and transportation” using ZigBee. The system displayed these raw values on an OLED screen; however, they did not offer any analysis. In addition, the acceleration was measured only when the user pressed a button.

Banerjee et al. [138] proposed a prototype for monitoring agricultural crops. The measured elements were humidity and temperature (DHT22), shock, carbon monoxide (CO) concentration, fire, and movement. Battery life was not considered, and measurements and messages were obtained every 30 min using a Wi-Fi connection; this is a very energy-consuming protocol. Measurements were sent to the user via email or SMS. No analysis was offered to simplify the user's task; however, in the future, the authors plan to detect theft using machine-learning techniques.

Dal-uyen et al. [139] developed a system with hardware components available in the Philippines to ensure safe storage by continuously monitoring temperature and relative humidity (seven SHT21 sensors); these sensors allow the nondestructive determination of moisture content using the modified Chung-Pfost equation. Five SHT21 sensors were installed inside the grain mass: one SHT21 in the aeration chamber and one outside. The moisture content was controlled using an aeration fan and a heater. The system was evaluated in a laboratory-scale silo (100 kg capacity) and compared with a control silo (100 kg capacity). The moisture content was determined using a moisture meter. Martyn et al. [143] presented QCONTROL, which is a wireless system for temperature and humidity monitoring, to reduce the cost of installation and operation of cable thermometers; this system comprises a stand-alone sensor (temperature and humidity) to be installed anywhere when desired, such as the outlet of the drying chambers and wireless rods with temperature sensors for grain temperature monitoring. Furtado et al. [140] presented and tested a system to control and monitor the drying process; however, the focus was also on cost. Román and Hensel [148] proposed a low-cost, low-power microcontroller humidistat to control the operation of a DC fan powered by a photovoltaic system for small storage in remote areas. The system cost was less than $US10 and they tested it with a Gombisa (a small traditional Ethiopian storage unit). Li et al. [142] proposed and tested a smart cooling ventilation system in field silos to optimize it with elaborate models; however, information about the control system, which includes the microcontroller and the human interface was minimal.

Xiaodong et al. [153] presented a system based on the S3C6410X and ARM-Linux embedded operating system to monitor environmental parameters such as grain temperature, humidity, and video images of the granary. However, a video of the granary seems incompatible with the embedded system during the grain storage period (owing to power and data consumption). Xu and Huang [159] proposed a monitoring system based on Raspberry Pi. It uses sensors to monitor the temperature, humidity, and oxygen concentration in the granary, use cameras to capture the pests in the grain pile, and take photo of the pests. Users can view sensors' data through Web or App and receive warning information from the system to achieve the purpose of real-time monitoring and warning. The Raspberry Pi collected data every 10 s. This was not compatible with a battery powered system.

Parvin et al. [147] proposed grain storage monitoring models optimized for the number and positioning of the nodes; they did not specify a precise architecture of a system but only provided some references for sensors such as DS18B20 (temperature sensor), SHT75 (temperature and humidity sensor), or SDP610 (pressure sensor). Although they did not offer telecommunication technology, their modelling focused on a network with nodes that transmitted information between them to a gateway. Unfortunately, these optimizations were not accompanied by a real system to experimentally confirm them.

Srilakshmipathy et al. [150] proposed a system that monitored only temperature and humidity. The system was powered by AAA batteries. An analysis of the consumption was proposed. The system communicated Lora on a Node-Red dashboard and the transmitter node was successfully tested inside a silo. The proposed system achieved long range wireless data transmission with range up to 500m with high accuracy, low cost, and low power consumption of 0.045W of each transmitter node.

Some studies selected programmable logic controllers (PLC) as used in industrial factories; e.g., a Siemens PLC [149]. Practice showed that the distributed monitoring-control system held various advantages of strong reliability, high stability, convenient operation and intuitive interaction. Zhao et al. [154] presented a temperature and humidity monitoring system based on an optical fiber sensor system designed for large granaries. They used the principle of monitoring changes in a fiber Bragg grating for temperature and humidity sensors and compared their new technology with a traditional sensor system in an experiment on a 2700-ton granary in China.

#### 5.3.5 Multi-sensor environmental monitoring systems

Table 2 shows comparison of embedded multi-sensor systems that monitor multiple grain storage environment quantities published between 2016 and 2023. Therefore, systems that do not measure at least two parameters (including temperature) were excluded. Thus, connected traps alone or with video/photo surveillance systems were not included.

Note that none of the publications considered in Table 2 mention the possible problems or precautions taken in terms of cybersecurity. These issues are addressed in smart agriculture in a more global manner but not in grain storage; this may be unfortunate because potential attacks can impact the food industry.

Only five publications analyzed the power consumption of their devices; two proposed a battery power supply without any analysis of the power consumption. However, interest in a system communicating via a wireless protocol is limited when the power supply is wired or when the battery must be changed frequently. Further, the choice of communication protocols does not always result in low energy consumption. Ten publications used Wi-Fi as a communication protocol; however, it is considerably energy-consuming (its interest lies in the high data rates allowed). Four publications proposed using Zigbee for silos and granaries; this seems judicious because Zigbee is a mesh protocol. This protocol is interesting when many devices are present and fixed in a restricted space, as is the case with a silo or granary. Only Mihajlo et al. [144] used an LPWAN protocol called NB-IoT. Only Srilakshmipathy et al. [150] used LoRa. None of them used other known LPWAN protocols such as Sigfox.

Further, most of these systems have been used for grain storage in silos or granaries. Only two studies focused on monitoring hermetic storage. Kumar et al. [26] proposed a system for modifying the atmosphere using gas sensors, and the proposed system was equipped with a CO_2_ sensor (that measured up to 100 % CO_2_) and a pressure sensor. This system seems to be suitable for a carbon dioxide-enriched atmosphere because of the measurement range of the sensor. This system is connected to an external power supply, and it is not suitable for small hermetic storage. Special attention must be focused on sealing when the system requires holes to pass through the container.

Most controllers and sensors are relatively large in size; for example, seven publications used Arduino Due or Mega Controllers. The most commonly used humidity and temperature sensors are DHT22 and DHT11, which are bulky and energy-consuming. The interest in these sensors arises only from their ease of integration and low cost. The system proposed by Wang et al. [151] is the only system that offers shock monitoring. Indeed, for storage that can be moved (such as big-bags, containers, etc.) or hermetic storage (bags, silo-bag, etc.), it is interesting to detect shocks that can damage the container or even the contents. However, measurement and data transmission via Zigbee are not automatic. Each measurement is performed by manually pressing a button via a digital interface, which is of limited practical interest.

### 5.4 Summary

Grain storage monitoring is part of smart agriculture. As discussed in this section, many companies now offer grain-storage monitoring systems; however, most of these systems only measure temperature and humidity, and they do not analyze these values. Further, there are few systems designed for storage other than silos, bins, granaries, and silo bags. Based on the proposed comparison, even experimental systems focus on these types of storage; these systems neglect modified atmospheric storage and associated gas sensors (except for some publications).

## 6 Conclusion and prospects

Advances in electronics and digital technology have opened new perspectives in the food storage sector, and new solutions facilitate storage monitoring using systems comprising wireless sensors with greater information processing capabilities. These systems go beyond simple measurements by offering detailed data analysis to simplify storage management, optimize ventilation operations for silo storage, or monitor phosphine levels during fumigations. However, all existing industrial systems and research propose actions based on exceeding thresholds. Further, the comparisons presented in Table 2 indicate that the optimization of energy consumption is not considered. Therefore, many scientific publications proposing battery-powered systems use energy-intensive communication protocols such as Wi-Fi. The selected solution uses a battery with a sufficiently high energy capacity, and the controllers and sensors are relatively large (e.g., Arduino controllers and DHT22 or DHT11 sensors). Wireless communication devices are bulky and expensive, and are not well suited for small storage volumes such as bags under one ton. Moreover, cybersecurity issues are being addressed for “smart agriculture,” although this is not the case for its application to grain storage. Silo storage in ambient air (with ventilation) is the most common storage solution, and thus, monitoring systems are often designed only for this type of storage. Storage in silo bags is considerably risky for grain quality preservation. Therefore, some systems have been developed to limit the risk of losses in silo bags by generating warnings as soon as possible in the case of possible deterioration. The evolution of flexible hermetic containers and past research on modified/controlled atmosphere technology has allowed new companies to offer safe storage without chemical treatments. The advent of silo bags, PICS, and other hermetic storage systems is one example; unfortunately, few publications have reported the continuous monitoring of storage in either an artificially oxygen-depleted atmosphere (whether by nitrogen injection or by creating a partial vacuum) or in a CO_2_-enriched atmosphere. However, shocks, rodents, or even certain insects can still continue to perforate some liners and degrade the modified atmosphere, and thus, the safe storage time remains uncertain.

Given this context, there is indeed a real need to develop a monitoring system for artificially modified atmospheres. To this end, a system that conducts an analysis of the parameters (at least humidity, temperature, and gas concentration) of the modified atmosphere of small hermetic containers should be used to help the storage manager make decisions. This system must include appropriate models (thermal models, CO_2_ adsorption, etc.) and machine learning methods to predict variations in environmental conditions and determine the evolution of storage quality and safe storage time. Beyond this analysis, it is also necessary to optimize the system in terms of battery consumption, volume, and cost. Optimizing battery consumption will help minimize battery capacity and contribute to the miniaturization of the device. Further, the electronics of each hermetic container must be composed of low-cost sensors to be compatible with this type of storage; the design of such a system adapted to hermetic big-bag storage with a modified atmosphere enriched in carbon dioxide will be considered in future work.

## CRediT authorship contribution statement

**Louis Labrot–Rhodes:** Writing – original draft, Investigation. **Eric Campo:** Writing – review & editing, Validation, Supervision. **Pierre Poujaud:** Supervision, Funding acquisition.

## Data availability statement

No new data was generated for the research described in the article.

## Ethics declaration

Review and/or approval by an ethics committee as well as informed consent was not required for this study because this article did not involve any direct experimentation/studies on living beings.

## Declaration of competing interest

The authors declare that they have no known competing financial interests or personal relationships that could have appeared to influence the work reported in this paper.
